# The effect of spiritual support on life satisfaction in Iranian patients with gastrointestinal cancer

**DOI:** 10.3389/fpsyg.2024.1378419

**Published:** 2024-05-22

**Authors:** Sajad Hamidi, Naeimeh Seyedfatemi, Zahra Abbasi, Fatemeh Ebrahimpour

**Affiliations:** ^1^School of Nursing and Midwifery, Iran University of Medical Sciences, Tehran, Iran; ^2^School of Nursing and Midwifery Shahid Beheshti, University of Medical Sciences, Tehran, Iran

**Keywords:** spiritual support, gastrointestinal cancer, life satisfaction, cancer, Iranian

## Abstract

**Background:**

The diagnosis and treatment of gastrointestinal cancer not only impose significant physical challenges but also deeply affect patients emotionally and psychologically, significantly influencing their overall quality of life. Among the various factors that can positively impact life satisfaction in individuals facing gastrointestinal cancer, spirituality emerges as a crucial aspect. This study aimed to determine the effect of a spiritual intervention on life satisfaction in patients with gastrointestinal cancer.

**Methods:**

This quasi-experimental study was conducted with two groups of 85 gastrointestinal cancer patients from two major hospitals in Tehran. The intervention group received spiritual support through social media for six sessions over three weeks to learn how to effectively improve their spiritual state. The control group received routine medical visits and care. The Satisfaction with Life Scale (SWLS) was used before and after the spiritual support in both the intervention and control groups. The research was approved by the institutional ethics committee.

**Results:**

In the pre-test stage, there was no significant difference in average life satisfaction between the intervention and control groups (*t* = 1.887, *d* = 0.30, *p* > 0.05). However, positive changes occurred in the post-test stage. Notably, the disparity in average life satisfaction between the intervention and control groups was significant in the post-test stage (*t* = 13.118, *d* = 0.95, *p* < 0.01). Furthermore, the result showed that the changes in life satisfaction in the intervention group were statistically significant (*t* = 11.854, *d* = 0.84, *p* < 0.001). Changes in life satisfaction in the control group were not statistically significant in the pre-test stage compared to the post-test stage (*t* = 1.113, *d* = 0.10, *p* > 0.05).

**Conclusion:**

The results can guide health care providers in dealing with the problems of cancer patients. Assessing patients’ spiritual needs and empowering them to promote their spiritual recovery and find meaning in their suffering can lead to improved quality of life and satisfaction with holistic care.

## Introduction

Gastrointestinal cancer is one of the most common cancers in different societies. In the United States, it is the second most common non-skin cancer after prostate. By 2040, projected shifts in age demographics and population growth indicate a significant surge in the global incidence of gastrointestinal cancers. It’s estimated that new cases will rise by 58%, reaching 7.5 million, while deaths from these cancers are expected to increase by 73% to 5.6 million (Arnold et al., 2020). Gastrointestinal cancers are the leading cause of cancer death in both men and women in Iran, as most patients are diagnosed at an advanced stage. The 5-year survival rate for patients with gastrointestinal cancer is estimated to be less than 25% (Farmanfarma et al., 2020). The number of new cancer cases in Iran is projected to increase from 112,000 recorded cases in 2016 to an estimated 160,000 in 2025. Stomach cancer was the most common cancer in Iran in 2016 and is predicted to remain the leading cancer nationally in 2025 (Roshandel et al., 2021). Cancer is a life-threatening disease that affects all aspects of life and poses various biological and psychological challenges for patients and their families. The disease causes a mismatch between individual preferences and needs, leading to an unsatisfactory life for cancer patients (Seiler and Jenewein, 2019).

Life satisfaction, a dynamic and multidimensional measure of general well-being, serves as a fundamental indicator of an individual’s subjective evaluation of their quality of life. Life satisfaction serves as a predictor of various outcomes, including mental health, physical health, and social functioning, underscoring its significance as a key determinant of overall quality of life. Alongside happiness and subjective well-being, life satisfaction constitutes one of the three primary indicators of personal well-being, offering insights into individuals’ perceptions of their life’s overall fulfillment and contentment (Diener et al., 1985, 2018). Life satisfaction has been used as a measure of recovery from illness and as a compatible indicator of new life: when patients are more satisfied with their lives, the symptoms of illness are less severe. Life satisfaction is associated with disease acceptance and coping strategies (Polański et al., 2020). Life satisfaction leads to an improved quality of life, improved ability to cope with stress, a positive outlook on life, and a reduction in psychological problems in cancer patients. The low level of life satisfaction has several consequences, including the exacerbation of the inappropriate clinical situation of chronic diseases (Rosella et al., 2019). It seems that spirituality is a necessary factor for achieving and protecting life satisfaction (Carranza Esteban et al., 2021). Spirituality is a dimension of a person’s relationship and integrity with the world. Relationships and integrity give hope and meaning to people and take them beyond time, place and material interests (Poorakbaran et al., 2018).

Spirituality is a human capacity that provides coping and problem-solving strategies to create a sense of meaning in the face of confusion and adversity. Internationally, the importance of patients’ spiritual needs over material needs has been emphasized. Spiritual care is considered to be one of the primary responsibilities of nurses (Harrad et al., 2019).

Apart from the physical hurdles, cancer patients often face reduced quality of life and survival because of psychological distress, such as spiritual crises, anxiety, and depression. Research has shown that spiritual intervention can alleviate anxiety and depression, leading to an improved overall quality of life and better adjustment to cancer (Xing et al., 2018). Nevertheless, despite their significance, these interventions are still inadequately implemented. Rouholamini et al. (2017) and Kamari and Fooladchang (2016) found that spiritual teachings in Iran play a positive role in life satisfaction. On the other hand, Ahmadi and Noormohammadi (2017) found that spiritual teaching did not affect quality of life in southeastern. Furthermore, in a literature review, Gonçalves et al. (2017) reported that the implementation of religious and spiritual interventions has little impact on improving the quality of life of patients suffering from cancer as a chronic disease.

One of the roles of the nurse’s psyche is to educate. The nurses’ psyche seeks to provide comprehensive interventions for cancer patients regarding spiritual care (Lavorato Neto et al., 2018). Spirituality is classified as one of the determining factors of people’s health, in addition spiritual beliefs shoed several improving effects on peace of mind and life satisfaction among them (Carranza Esteban et al., 2021). Given the conflicting results in the literature and the need to investigate the influence of this type of intervention in patients with gastrointestinal cancer, the current study aims to answer the following question Does spiritual education influence life satisfaction in patients with gastrointestinal cancer?

## Method

This study follows an effect evaluation approach, involving two groups with pre-test and post-test assessments, conducted at two hospitals affiliated with the Iran University of Medical Sciences. These hospitals are primary centers for oncology services in Iran. In this study, individuals diagnosed with gastrointestinal cancers constituted the research community. Information about individuals with gastrointestinal cancer was obtained continuously from the mentioned hospitals and visits to inpatient wards in two periods: from March 5, 2021, to March 18, 2021, and from June 1, 2021, to July 1, 2021 (the reason for the interval in sampling was coronavirus peak and prohibition of the attending of ordinary people to medical centers). Additionally, data on hospitalized patients diagnosed with gastrointestinal cancer were obtained from the medical records unit from September 22, 2020, to July 1, 2021. Due to the pandemic situation, the phone was used administering the intervention and completing the questionnaire. The intervention group received spiritual teaching for six sessions over three weeks and the control group received routine medication in the medical centers.

The sample size was estimated to be a minimum of 76 participants in each group, with a confidence level of 95% and power of 80%, and was calculated to be 85, with a sample attrition rate of 10%. According to Steger et al. (2006), the standard deviation (SD) was estimated to be 6.6 in each group. Inclusion criteria were age between 18 and 65 years, confirmed diagnosis of gastrointestinal cancer (including oesophageal, gastric, colorectal, small bowel, pancreatic, liver and gallbladder cancers) by a specialist, and having a smartphone. In addition, having WhatsApp software and being able to use it, and not having a chronic disease (heart disease and diabetes) according to the patient’s comments were other inclusion criteria. Exclusion criteria were leaving the educational group on WhatsApp and lack of cooperation from the patient in presenting the task due to absence from the study or death.

After receiving the ethical code (IR.IUMS.REC.1399.999) from the research assistant of Iran University of Medical Sciences, a sample was taken from Firouzgar and Rasoul Akram hospitals in Tehran. The researcher contacted the patients after obtaining their information from the medical records department of the hospitals and obtained their informed consent after explaining the purpose of the study. The participants were then contacted to arrange prior appointments and to coordinate data collection. The participants completed a questionnaire comprising 15 questions within a 15-min timeframe. In addition, participants were informed that the telephone calls would be recorded and transcribed afterwards. If the patient was unable to complete a task or call, the rest of the questionnaire was completed at another time. 85 patients were in the experimental group and 84 were in the control group. On the other hand, it is necessary to explain that 3 groups (30, 30, and 25) were formed, and these groups were made up of participants from the experimental group. WhatsApp, as a popular social media in Iran, offers easier use and better facilities than other messaging services, so three WhatsApp groups with spiritual teaching titles were created (two groups of 30 people and one group of 25 people). The research team (psychiatric nurse and doctor of nursing) joined these groups along with the intervention group to monitor the implementation process. Spiritual education was then given to the patients in six sessions (twice a week for 30 min) over three weeks. This period was programmed in such a way that the patients could receive the teaching content in a short time and achieve a higher educational efficiency. The teaching took the form of uploading video films made by the researcher.

During the educational content development phase, validated databases were searched using the keywords education, spirituality, life satisfaction, cancer and gastrointestinal cancer. Several articles were extracted, including programmes that teach spiritual principles to patients. In addition, the texts of the Spiritual Guidance Book by Bolhari et al. (2019), the Medical Spiritual Teaching by Vaziri (2017), and the American National Cancer Institute guideline (PDQ Supportive and Palliative Care Editorial Board, 2002) were used as the content of the teaching. The extracted content was then classified and tested. The validity of the booklet was confirmed by five faculty members of Nursing Faculty in Iran as masters of teaching spirituality. In addition, Camtasia software was used to prepare video content. Interventions backed by at least three faculty members were retained, while those supported by fewer than three were disregarded. Questions from all sessions were uploaded to the chat group and answered by the research group. All educational videos had a final question and participants were asked to send the answer to the researcher privately on WhatsApp within 24 h. The researcher had to make sure that all participants observed the content. The only educational task was to answer the final question in each video. One month after the end of the intervention, a post-test was administered to both groups. After the post-test, all educational content was uploaded as educational videos on WhatsApp for the control group. It should be noted that all members of the control group were receiving their usual medication at the medical centers during the intervention, and the pre-and post-tests were taken by telephone. One member of the intervention group was excluded from the spirituality teaching during the study and was excluded from the study. The educational sessions are summarised in Table 1 with their original content.

**Table 1:** The content of the teaching of spirituality.

Sessions	Intervention techniques	Sessions title	Teaching content
Session 1	Psychoeducation	How to teach on WhatsApp as a social media, defining the concept of spirituality, and the meaning of life	Introduction of the teachers, definition of the content and application of WhatsApp as a social media, presenting information about the reasons for creating a group chat; definition of spiritual life, the meaning of spirituality and characteristics of spiritual people, the meaning of life and methods of giving meaning to life.
Session 2	Psychoeducation	Defining the concept of introversion, self-awareness, and the importance of spirituality in self-awareness	What is introversion? What is self-awareness? Why is self-awareness important? What are different types of self-awareness? What is spiritual self-awareness? What are the effects of spiritual self-awareness?
Session 3	Psychoeducation	Sources of fear, stress, and coping with them	What are the sources of anxiety and stress, how to deal with anxiety and stress, what is anxiety and stress, what are the methods to get peace of mind, and what is the effect of trust in getting peace of mind? What are the cognitive tools of trust, results and consequences of trust?
Session 4	Psychoeducation	Efficient safeguarding of valuable heritage	What is infinite? What does kindness mean? Definition of meditation. The definition of patience. How do individuals behave in the face of problems? Beliefs undermining patience and methods to enhance it.
Session 5	Psychoeducation	Comprehending forgiveness and resolving anger	How to resolve anger? What does forgiveness mean? Name the steps before and after forgiveness. Benefits and consequences of forgiveness
Session 6	Psychoeducation	Addressing a problem using a spiritual perspective	Assessment of clients’ familiarity with the concept of problem-solving and the stages of problem-solving using a spiritual approach (What is the precise definition of a problem? How are the stages of problem-solving without a spiritual approach and with the help of a spiritual approach?)

A demographic information form was used to collect data and measure the variables of age, gender, marital status, economic status, employment status, and educational level, type of cancer and stage of cancer. The Satisfaction with Life Scale (SWLS) by Diener et al. (1985) was used, with five items and responses on a 7-point Likert scale. Scores 5–9 indicate extremely unhappy, scores 10–14 indicate unhappy, scores 15–19 indicate somewhat unhappy, score 20 indicates neutral, scores 21–25 indicate somewhat happy, scores 26–30 indicate happy, and scores 31–35 indicate extremely happy. For all items, a score of 7 indicates extremely happy and a score of 1 indicates extremely unhappy. The range of life satisfaction scores was 5–35, so the higher the individual’s score on this scale, the more satisfied the participant was with life (Diener et al., 1985). The designer has reported 87% for Cronbach’s alpha coefficient scale. In this study, the content validity of the instruments was verified by five faculty members of nursing department of Iran University of Medical Science. In addition, the reliability of the measure was verified using Cronbach’s alpha coefficient scale through the participation of 15 clients suffering from gastrointestinal cancer who were not members of the research sample. The obtained coefficient was calculated as 0.86% and SPSS-16 software was used for data analysis. Cronbach’s alpha reliability in the pre-test of the intervention and control groups was 0.92 and 0.78, respectively. and in the post-test of the intervention and control groups, 0.79 and 0.88, respectively. Descriptive statistics, including frequency distribution and numerical indicators, and inferential statistics, including chi-square tests, independent-Sample *t*-test to compare the average life satisfaction of the intervention and control groups by pre-test and post-test phases (inter-group comparison), and paired samples dependent t-test to compare the average in life satisfaction between pre-test and post-test stages by intervention and control group (intra-group comparison) and were used to report data.

## Findings

The individual social profiles of the patients in the control and intervention groups are shown in Table 2. In terms of disease characteristics, most patients in the control group (57.6%) and intervention group (58.3%) had a family history of gastrointestinal cancer. Most patients in both groups had colorectal, stomach or rectal cancer. The cancer was reported in the second stage in patients in the control (58.8%) and intervention (51.2%) groups.

**Table 2:** Frequency distribution of the individual profiles as the research participants in the control and intervention groups.

Personal information		Control		Intervention		Test result
		*n*	%	*n*	%	
Gender	Male	53	62.4	47	56	$\chi^{2}=0.716$ df = 1 *p* = 0.397
	Female	32	37.6	37	44	
	Total	85	100	84	100	
Age (year)	Less than 30	8	9.4	6	7.1	$\chi^{2}=0.534$ df = 3 *p* = 0.929
	30–39	27	31.8	20	23.8	
	40–49	18	21.2	30	35.7	
	50–59	21	24.7	21	25	
	More than 60	11	12.9	7	8.3	
	Total	85	100	84	100	
	Mean ± SD	44.35 ± 11.21		44.2 ± 10.65		
	Min-max	25–64		25–64		
Marital status	Single	14	16.5	8	9.5	$\chi^{2}=2.321$ df = 3 *p* = 0.346
	Married	64	75.3	72	85.7	
	Divorced	5	5.9	2	2.4	
	Widowed	2	2.4	2	2.4	
	Total	85	100	84	100	
Number of children	0	10	14.1	9	11.8	$\chi^{2}=$ 1.275df = 3 *p* = 0.735
	1	13	18.3	10	13.2	
	2	18	25.4	24	31.6	
	More than 3	30	42.3	33	43.4	
	Total	85	100	84	100	
Economic status	Weak	25	29.4	31	36.9	$\chi^{2}=$ 4.647df = 2 *p* = 0.098
	Medium	48	56.5	49	58.3	
	Good	12	14.1	4	4.8	
	Total	85	100	84	100	
Employment	Employee	18	21.2	19	22.6	$\chi^{2}=$ 5.525df = 3 *p* = 0.137
	Self-Employment	30	35.3	22	26.2	
	Retired	21	24.7	15	17.9	
	Unemployed	16	18.8	28	33.3	
	Total	85	100	84	100	
Education	Less than a diploma	20	23.5	28	33.3	$\chi^{2}=$ 2.043df = 2 *p* = 0.36
	Diploma	22	25.9	20	23.8	
	College	43	50.6	36	42.9	
	Total	85	100	84	100	

Life satisfaction was examined in the pre-test stage between the two groups, which showed no significant difference (*p* > 0.05). Based on the results of the independent *t*-test, there was no significant difference between the intervention and control groups in the average life satisfaction in the pre-test stage (*p* > 0.05), but there were positive changes in the post-test stage, and the difference in the average life satisfaction in the post-test stage between the intervention and control groups was significant (*p* < 0.001) (Table 3). Furthermore, the result of the paired sample *t*-test showed that the changes in life satisfaction in the intervention group were statistically significant (*p* < 0.001). Changes in life satisfaction in the control group were not statistically significant in the pre-test stage compared to the post-test stage (*p* > 0.05) (Table 4).

**Table 3:** Frequency distribution, mean ± SD of life satisfaction for the research participants among the control and intervention groups in their pre-test and post-test stages (inter-group comparison).

Life satisfaction (5–35)*	Pre-test (totals)		Post-test (totals)	
	Control	Intervention	Control	Intervention
Strongly disagree (5–9)	32	9	12	1
Disagree (10–14)	31	29	42	18
Slightly disagree (15–19)	19	21	16	33
Neither agree nor disagree (20)	0	2	7	7
Slightly agree (21–25)	3	20	8	23
Agree (26–30)	0	3	0	2
Strongly agree (31–35)	0	0	0	0
Total	85	85	85	84
Mean ± SD	13.30 ± 4.11	14.96 ± 5.48	13.95 ± 4.31	18.92 ± 4.09
Min-max	6–23	75–30	7–24	9–27
Result of independent	*t* = 1.887, *p* > 0.05		*t* = 13.118, *p* = 0.001	
*t*-test	*d* = 0.30		*d* = 0.95	

*d* = Cohen’s d. * Mean score range of life satisfaction.

**Table 4:** Frequency distribution, mean ± SD of life satisfaction for the research participants among the control and intervention groups in their pre-test and post-test stages (intra-group comparison).

Life satisfaction (5–35)*	Control				Intervention			
	Pre-test		Post-test		Pre-test		Post-test	
	*n*	%	*n*	%	*n*	%	*n*	%
Strongly disagree (5–9)	32	37.6	12	14.1	9	10.5	1	1.2
Disagree (10–14)	31	36.5	42	49.4	29	35.2	18	21.4
Slightly disagree (15–19)	19	22.4	16	18.8	21	24.7	33	39.3
Neither agree nor disagree (20)	0	0	7	8.2	2	2.6	7	8.3
Slightly agree (21–25)	3	3.5	8	9.4	20	23.5	23	27.4
Agree (26–30)	0	0	0	0	3	3.5	2	2.4
Strongly agree (31–35)	0	0	0	0	0	0	0	0
Total	85	100	85	100	85	100	84	100
Mean ± SD	13.30 ± 4.11		13.95 ± 4.31		14.96 ± 5.48		18.92 ± 4.09	
Min-max	6–23		7–24		5–30		9–27	
Result of the paired	*t* = 1.113, *p* > 0.05				*t* = 11.854, *p* < 0.001			
*t*-test	*d* = 0.10				*d* = 0.84			

*d* = Cohen’s d. * Mean score range of life satisfaction.

## Discussion

This study aimed to determine the effect of spiritual teaching on life satisfaction in patients with gastrointestinal cancer. The study showed that spiritual teaching could create a significant difference in the mean score of life satisfaction among patients in the intervention group. Spirituality can be an important source of comfort for patients suffering from chronic illnesses, including cancer. The implementation of cognitive-behavioural, patient-centred and educational interventions, such as spiritual teaching, through the creation of a safe environment with the removal of environmental stressors, regular sleep habits and the prevention of fatigue, leads to an increase in physical health. This will ultimately improve the patient’s quality of life and life satisfaction (Navidian and Bahari, 2008). The study by Kazemi et al. (2018) was in line with the current study findings. The patients in his study suffered from breast cancer in Iran while the present research studied patients suffering from gastrointestinal cancer. The patients in the study by Kazemi et al. in Iran received religious and spiritual counseling and the program was carried out for 10 weeks. On the other hand, patients in the current study received six sessions over three weeks of virtual spiritual education. Considering the different nature of the intervention methods and the different presentation of the topics to the patients in both studies, it still seems that spirituality is effective in increasing life satisfaction in patients suffering from various cancers. The study of Rudaz et al. (2019) was consistent with the present study. Spiritual teaching for patients suffering from cancer in America was carried out through the presentation of spiritual advice, which led to an improvement in the mental well-being of the participants. In addition, daily remembrance of spiritual experiences and increasing the level of implementation of religious/spiritual exposure improved the life satisfaction of the patients. On the contrary, the life satisfaction of these patients was at a very inappropriate level before the intervention (Rudaz et al., 2019).

In addition, Wu and Koo (2016) in Taiwan reported that spiritual intervention improved degrees of expectancy, life satisfaction, and spiritual well-being among elderly people suffering from Dementia disease during a six-week intervention.

It seems that an improvement in life satisfaction was possible in the group of elderly people considering the relationship between life satisfaction, spiritual well-being and religious functions of spirituality. Therefore, regarding the congruence between the mentioned study and our research, it can be claimed that patients suffering from chronic diseases can experience an improvement in life satisfaction by having a relationship between spirituality and its use in life, regardless of the type of disease. Furthermore, patients suffering from gastrointestinal cancer can also benefit from this relationship.

Abdi et al. (2019) conducted a study in Iran to investigate the effect of a spiritual intervention on life satisfaction in elderly people suffering from heart failure. Their study found that the effect of spiritual teaching on the intervention group after the intervention was consistent with our study. The intervention implemented for the intervention group had a religious-spiritual programme designed based on the rules of Islam and Shiism and lasted for six sessions of 30–45 min (Abdi et al., 2019). It seems that an improvement in life satisfaction was possible in the group of older people, considering the relationship between life satisfaction, spiritual well-being and religious functions of spirituality. Therefore, regarding the congruence between the mentioned study and our research, it can be claimed that patients suffering from chronic diseases can experience an improvement in life satisfaction by having a relationship between spirituality and its use in life, regardless of the type of disease. Furthermore, patients suffering from gastrointestinal cancer may also benefit from this relationship.

Abdi et al. (2019) conducted a study in Iran to investigate the effect of a spiritual intervention on life satisfaction in elderly people suffering from heart failure. Their study found that the effect of spiritual teaching on the intervention group after the intervention was consistent with our study. The intervention for the intervention group consisted of a religious-spiritual programme based on the rules of Islam and Shiism and lasted for six sessions of 30–45 min (Riklikienė et al., 2020).

It can be argued that the reasons for the incompatibility between the present study and the study by Ahmadi et al. were the patients suffering from different types of cancer as the research population, the random sampling method and the different inclusion criteria (suffering from cancer for at least one year). Furthermore, the face-to-face teaching by the psychologist, the different educational content and follow-up, and the differences in the variables studied were other differences of this study compared to the study by Ahmadi et al. On the other hand, this study investigated patients suffering from gastrointestinal cancer using non-probability sampling and virtual teaching by the psychiatric nurse at different stages. Considering the results of the current study, it can be stated that spiritual teaching can lead to an improvement in life satisfaction among cancer patients.

According to the results, the life satisfaction scores of patients in the control group increased at the post-test stage compared to the pre-test stage, but it was not a significant increase. Although the physical distance between the control and intervention groups prevented the exchange of information, it seems that the control group received spiritual teaching from other sources, including routine teaching in the medical centers. It seems that the provision of routine care by the medical centers studied was not effective in improving the life satisfaction of gastrointestinal cancer patients in the present study. Therefore, it can be recommended that medical centers continue to provide routine care for patients with gastrointestinal cancer and try to help them recover from the disease. In this regard, Jin and Lee (2019) reported a non-significant change in life satisfaction among the patients suffering from cancer in the control group receiving routine care in hospitals in South Korea. The study by Jin and Lee is in congruence with the present research. Both studies are similar due to lacking attention, following the medical centers in terms of life satisfaction status among patients with cancer, and meeting their needs using appropriate teaching in line with improving their level of life satisfaction. The study by Mi-kyeong et al. was in line with the current study as well. Mi-kyeong study indicated the absence of routine teachings effects on life satisfaction improvement among elderly patients suffering from gastrointestinal cancer in medical centers in South Korea (Mi-kyeong et al., 2019). Patients in the current research had a younger age range than the mentioned study. Thus, since younger age is along with a low possibility of suffering from debilitating diseases simultaneously, it might intervene in life satisfaction. Nevertheless, the results of both studies are similar and more studies are needed to be carried out in this field.

It seems that using spiritual topics for the studied population can improve life satisfaction by increasing awareness of spiritual doctrines and the method of using them in particular life conditions including cancer. Cancer creates feelings of agitation, depression, stress, despair, death, guilt, and worthlessness in people’s life. Besides, people’s mentality is influenced by complicated factors such as individual beliefs and culture (Riklikienė et al., 2020). Therefore, it seems that the population h studied in this research being under psychological tension effect, achieved life satisfaction improvement using spiritual teaching and a tendency to continue fighting against cancer.

The results of the aforementioned studies are compatible with the current study. Therefore, this training can adjust the satisfaction status of patients suffering from chronic diseases such as cancer, despite the differences in the studies on spiritual teaching, which are the result of cultural, ethnic, psychological and social factors. Nevertheless, the compatible studies mentioned with the current study revealed a two-way relationship between the degree of use of spiritual teaching and life satisfaction for all patients. It seems that more studies in this area should be carried out among patients suffering from gastrointestinal cancer, and the effect of spiritual teaching on life satisfaction can be judged decisively among this group of patients. Therefore, spiritual teaching may have positive effects on improving life satisfaction among patients with gastrointestinal cancer. In addition, the difference in the effectiveness of the patients’ spiritual beliefs in the two groups studied can be considered as another limitation. This study was carried out with a limited number of patients in two hospitals; therefore, its result cannot be generalised to the community of patients suffering from gastrointestinal cancer. The Covid-19 pandemic prevented the intervention from being carried out in a face-to-face manner, and finally it was carried out virtually, which was one of the innovations of this study in terms of the flexibility of this method.

## Conclusion

The current study showed that a spiritual teaching intervention can lead to a significant improvement in patients’ life satisfaction after a one-month follow-up. It seems that the patients who were referred to medical centers at that time could ask all their questions and get an appropriate answer. Considering the positive result of spiritual teaching on life satisfaction among patients suffering from gastrointestinal cancer, this study can be useful for this group of patients in many areas. The treatment staff, especially the nurses, need to have complete knowledge of the physical and mental condition of the patients, their problems, strategies, and functional programs to meet the patient’s needs. Psychiatric nurses can support cancer patients to improve their life satisfaction using spiritual concepts and definitions and spiritual-based educational programs.

Spiritual education in a virtual way can be an inexpensive strategy to prevent medical costs of mental disorders such as chronic diseases, to care for patients, and to rehabilitate their physical and mental aspects. This could include lectures, workshops, written materials, or discussions aimed at enhancing patients’ understanding of spirituality and its potential impact on their well-being. Spiritual counseling involves personalized support and guidance trained professionals provide to help individuals explore and address their spiritual concerns, questions, and experiences. This form of counseling aims to help patients find meaning, hope, and comfort in their spiritual beliefs, thereby potentially enhancing their overall well-being and life satisfaction. Clarifying the distinction between spiritual teachings/education and spiritual counseling can provide a comprehensive understanding of how different forms of spiritual support may impact the life satisfaction of Iranian patients with gastrointestinal cancer.

## Data availability statement

The original contributions presented in the study are included in the article/supplementary material, further inquiries can be directed to the corresponding author.

## Author contributions

SH: Writing – review & editing, Writing – original draft, Visualization, Validation, Formal analysis, Conceptualization. NS: Writing – original draft, Visualization, Project administration, Investigation, Conceptualization. ZA: Writing – original draft, Methodology, Formal analysis, Data curation. FE: Writing – original draft, Visualization, Project administration, Investigation.
